# Between-subject variability of muscle synergies during a complex motor skill

**DOI:** 10.3389/fncom.2012.00099

**Published:** 2012-12-28

**Authors:** Julien Frère, François Hug

**Affiliations:** ^1^Laboratory « Motricité, Interactions, Performance », University of MaineLe Mans, France; ^2^Laboratory « Motricité, Interactions, Performance », University of NantesNantes, France

**Keywords:** motor modules, muscle coordination, nonegative matrix factorization, motor primitives, electromyography, backward giant circle, gymnastics

## Abstract

The purpose of the present study was to determine whether subjects who have learned a complex motor skill exhibit similar neuromuscular control strategies. We studied a population of experienced gymnasts during backward giant swings on the high bar. This cyclic movement is interesting because it requires learning, as untrained subjects are unable to perform this task. Nine gymnasts were tested. Both kinematics and electromyographic (EMG) patterns of 12 upper-limb and trunk muscles were recorded. Muscle synergies were extracted by non-negative matrix factorization (NMF), providing two components: muscle synergy vectors and synergy activation coefficients. First, the coefficient of correlation (*r*) and circular cross-correlation (*r*_max_) were calculated to assess similarities in the mechanical patterns, EMG patterns, and muscle synergies between gymnasts. We performed a further analysis to verify that the muscle synergies (in terms of muscle synergy vectors or synergy activation coefficients) extracted for one gymnast accounted for the EMG patterns of the other gymnasts. Three muscle synergies explained 89.9 ± 2.0% of the variance accounted for (VAF). The coefficients of correlation of the muscle synergy vectors among the participants were 0.83 ± 0.08, 0.86 ± 0.09, and 0.66 ± 0.28 for synergy #1, #2, and #3, respectively. By keeping the muscle synergy vectors constant, we obtained an averaged VAF across all pairwise comparisons of 79 ± 4%. For the synergy activation coefficients, *r*_max_-values were 0.96 ± 0.03, 0.92 ± 0.03, and 0.95 ± 0.03, for synergy #1, #2, and #3, respectively. By keeping the synergy activation coefficients constant, we obtained an averaged VAF across all pairwise comparisons of 72 ± 5%. Although variability was found (especially for synergy #3), the gymnasts exhibited gross similar neuromuscular strategies when performing backward giant swings. This confirms that the muscle synergies are consistent across participants, even during a skilled motor task that requires learning.

## Introduction

Understanding how the central nervous system controls movement of the human body is a challenging question due to the biomechanical redundancy of the neuromusculoskeletal system, which is referred to as Bernstein's degrees of freedom problem (Bernstein, 1967). For example, at the neuromuscular level, the same movement can be performed by different muscle coordination strategies across trials (Torres-Oviedo and Ting, 2007) and/or between subjects (Ryan and Gregor, 1992; Hug et al., 2004). Low-dimensional modules formed by muscles activated in synchrony, referred to as muscle synergies, have been proposed as building blocks that may simplify the construction of motor behaviors (Ivanenko et al., 2003; 'Avella and Bizzi, 2005; Ting and McKay, 2007; Torres-Oviedo and Ting, 2007; Ting and Chvatal, 2010). The decomposition of multiple surface electromyographic (EMG) signals can be used to extract these synergies. This decomposition algorithm is based on two components: “muscle synergy vectors” which represent the relative weighting of each muscle within each synergy; and a “synergy activation coefficient” which represents the recruitment of the muscle synergy over time (Torres-Oviedo and Ting, 2007; Hug et al., 2011). Some previous research has proposed that temporal recruitment patterns are invariant while the weights can change across subjects/test conditions (Ivanenko et al., 2004, 2005; Cappellini et al., 2006; Dominici et al., 2011). Others have suggested that the muscle synergies are spatially fixed (i.e., muscle weightings are invariant) across subjects/test conditions while temporal recruitment patterns can change (Saltiel et al., 2001; Hart and Giszter, 2004; Torres-Oviedo and Ting, 2007; Hug et al., 2011; Safavynia and Ting, 2012). In line with this latter proposition, it has been shown during both postural (Torres-Oviedo and Ting, 2010) and locomotor tasks (Hug et al., 2011; Chvatal and Ting, 2012) that muscle synergy vectors (i.e., muscle weightings) are robust across various mechanical constraints allowing the temporal recruitment to vary according to the task demand. Moreover, altering the recruitment pattern of spatially fixed muscle synergies can produce different motor behaviors in animals (Cheung et al., 2005; Kargo et al., 2010).

As proposed by Safavynia et al. (2011), the acquisition of new motor skills can encourage the development of new muscle synergies, change the composition of existing synergies, and/or change their temporal activation. Through the process of learning, the modulation of the number and/or the composition of muscle synergies has been identified for postural tasks in humans (Asaka et al., 2008; Danna-Dos-Santos et al., 2008) and for reach-to-grasp tasks in rodents (Kargo and Nitz, 2003). Simultaneously with improving performance, the composition of the muscle synergies modulates toward consistent patterns across the animals, in terms of both synergy composition and temporal recruitment (Kargo and Nitz, 2003). Hug et al. (2010) reported similar muscle synergies among trained cyclists. However, one common feature of the movements studied in the aforementioned studies is that they can be considered as fundamental or basic movement skills (mainly locomotor and balance skills) and consequently all healthy subjects would be able to perform them with similar mechanical performance in terms of both kinematics and kinetics. As evidence, kinetic patterns in terms of both effective force and mechanical effectiveness are very similar between untrained subjects and trained cyclists (Sanderson, 1991; Mornieux et al., 2008).

The purpose of the present study was to determine whether experts exhibit similar neuromuscular control strategies during a complex motor skill. In other words, did the learning process necessary to perform this task led to similar muscle synergies or did each individual develop specific synergies related to their personal anthropometric, anatomical, or muscular characteristics? In order to answer these questions, we looked at a homogeneous population of nine experienced gymnasts performing giant swings on a high bar. This cyclic movement is interesting because it requires learning, as untrained subjects are unable to perform this task. As proposed by Wulf and Shea (2002), motor tasks can be qualified as “complex” if they cannot be mastered in a single session. Consequently, we considered the gymnastic backward giant swing on a high bar as a complex motor skill that would provide a contrast to fundamental motor skills such as balance, walking, or pedaling. For the purpose of this study, we used a non-negative matrix factorization (NMF) algorithm to identify muscle synergies from surface electromyographic recordings performed on 12 upper-limb and trunk muscles of the right side. In light of recent studies (Chvatal and Ting, 2012; Safavynia and Ting, 2012), we hypothesized that performing a complex motor performance would result from the recruitment of similar spatially fixed muscle synergies, which would be flexibly recruited over the giant swings, across all individuals.

## Materials and methods

### Participants

Nine gymnasts performing at national level (age: 19.8 ± 2.5 years, height: 171 ± 8 cm, body mass: 66 ± 8.1 kg) and with 14 ± 3 years of training experience participated in this study. They were informed of the purpose of the study and methods used before providing written consent. The local ethics committee (University of Nantes) approved the study, and all the procedures conformed to the Declaration of Helsinki (last modified in 2004).

### Protocol

**Figure 1:**
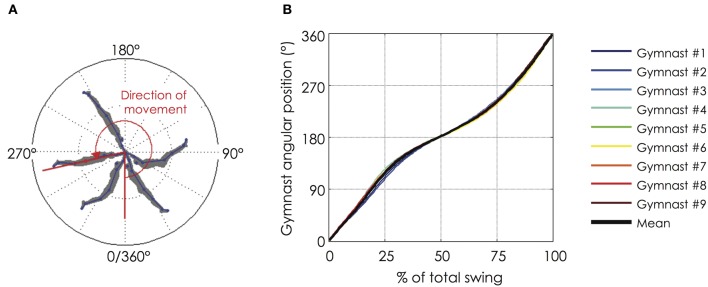
**Angular position of the gymnast during a backward giant swing. (A)** The angular position of the gymnast from the bar was defined as the angle formed by the axis linking the femoral trochanter (hip marker) with the bar and the vertical axis of reference (below the bar); **(B)** Evolution of the angular position of the gymnast as a function of the normalized time of the backward giant swing. The black bold line represents the mean value of the group while the thin color lines represent mean value among 9 successive giant swings for each gymnast. The gymnasts performed their backward giant swings in a very similar fashion (for more details see section “Mechanical Data”). The relationship between the angular position and the normalized time of the backward giant swing is not linear. The lower part (0–90° and 270–360°) is shorter than the upper part (90–270°) of the giant swing.

Participants were asked to perform two sets of 11–12-linked backward giant swings, with 3–5 min of rest period in-between. A giant swing was defined as a complete rotation of the subject around the high bar. In this study, we considered the beginning and the end of a giant swing as when the gymnast was in the vertical position under the bar (Figure 1). To manage a complete rotation around the bar, the gymnasts can vary their body length to account for the loss of velocity due to the effect of friction between the hands and the bar. The gymnasts extend their body away from the bar to lengthen his radius of gyration during the descent phase, and shorten their body in the ascent phase (Sevrez et al., 2009). To do this and in line with the recommendations from the point code of the International Gymnastic Federation, the elbow and knee joints should be maintained in extension and only flexion-extension of the shoulder and hip joints are authorized for varying the body length.

### Materials and data collection

#### Motion analysis

**Figure 2:**
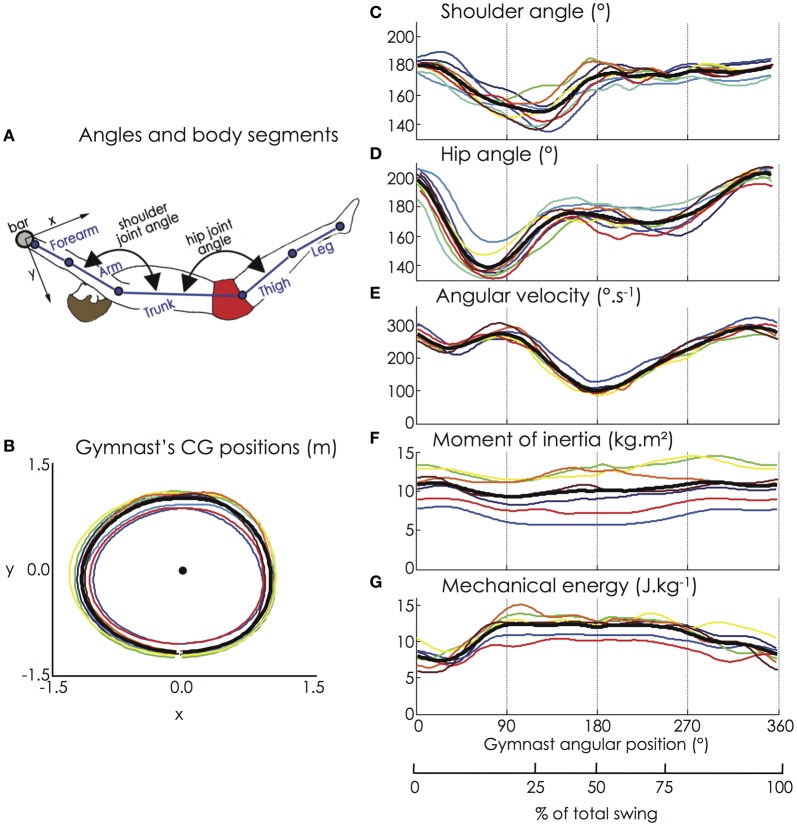
**(A)** Joint angles and body segments of the gymnast model; **(B)** Horizontal and vertical positions of the gymnasts' CG; **(C)** Flexion-extension of the shoulder joint; **(D)** Flexion-extension of the hip joint; **(E)** Angular velocity of the gymnast model (hip marker); **(F)** Moment of inertia, and **(G)** Mechanical energy of the gymnast model. The black bold line represents the mean value of the group while the thin color lines represent the mean value among 9 successive giant swings for each gymnast (for the color legend, see Figure 1). Except for the moment of inertia that exhibited a high interindividual variability due to large time shifts, all the biomechanical variables were similar among the participants.

The giant swings were recorded with a video camera (Casio Exilim EX-ZR100, Casio Computer Co. Ltd., Tokyo, Japan) in the main plane of movement (i.e., sagittal plane) with a sampling frequency of 120 Hz. The camera was placed along the longitudinal axis of the bar at a distance of 5 m and a height of 2.60 m, equivalent to the height of the bar from the landing mat. The placement of the video camera had to cover a sufficient range to record the entire body of the gymnast during the giant swing, with the high bar at the center of the field. The calibration square was 1 × 1 m and the origin of the inertial coordinate system was located at the center of the bar in its neutral position. The x-axis was defined as the horizontal axis in the main plane of movement. The y-axis was defined as the vertical axis. The angular position of the gymnast from the bar was defined as the angle formed by the axis linking the femoral trochanter (hip marker) with the bar and the y-axis of reference (below the bar). To reconstruct a multi-segment model of the gymnast, adhesive strips were placed on defined body locations for use as markers. The digitization of body marks was performed using Skillspector© software (Video4coach, Svendborg, Denmark) for the lower extremity (ankle, knee, and hip joints) and the upper extremity (wrist, elbow, and shoulder joints). The trunk was delineated by the shoulder and the hip. Thus, the model was composed of five segments (Figure 2): the arm, forearm, trunk, thigh, and leg. The masses and moments of inertia of different segments were calculated using an anthropometric table (de Leva, 1996), which was adjusted with consideration that the digitized model had one upper and one lower limb. Position data was smoothed using a 4th order low-pass Butterworth filter with a cut-off frequency of 5 Hz.

#### Surface electromyography

From the assumption of symmetry in the actions of both upper limbs during the execution of the elements on the high bar, and to avoid electrocardiogram artifacts, the activity of 12 muscles of the right side of the body was recorded: flexor digitorum (FD), short head of the biceps brachii (BBsh), long head of the biceps brachii (BBlh), lateral head of the triceps brachii (TB), clavicular (anterior) and scapular (posterior) parts of the deltoideus (DC and DS, respectively), upper part of the trapezius (TZ), latissimus dorsi (LD), sternocostal part of the pectoralis major (PM), rectus abdominis (RA), erector spinae at level of L4 (ES), and rectus femoris (RF). The surface EMG recordings were made using self-adhesive Ag/AgCl pairs of electrodes (Blue sensor N, Ambu, Denmark) with an inter-electrode distance of 20 mm (center-to-center). The electrodes were placed longitudinally with respect to the underlying muscle fiber arrangement (de Luca, 1997) and were located according to the recommendations of Surface EMG for Non-Invasive Assessment of Muscles (SENIAM) (Hermens et al., 2000) when available. For back muscles (TZ, LD, and ES), the electrodes position was according to de Sèze and Cazalets (2008). Skin was shaved and cleaned with alcohol and ether to minimize impedance before applying the electrodes. The wires connected to the electrodes were secured carefully with adhesive tape to avoid any movement-induced artifacts. Raw EMG signals were preamplified close to the electrodes (gain 375, bandwidth 8–500 Hz) at a sampling rate of 1000 Hz (ME6000, Mega Electronics Ltd., Kuopio, Finland). The EMG device was firmly attached on a belt during execution of the giant swings.

#### EMG-video synchronization

To synchronize the motion capture with the EMG recordings, percutaneous muscular stimulation (model DS7A, Digitimer Ltd., Letchworth Garden City, UK) was performed on the forearm muscles of the gymnast prior to and subsequent to each set of 12-linked giant swings. Both video and EMG were recorded when the stimulation was applied, bringing a brief artifact on the EMG signal of the FD muscle and lighting a LED in the field of the video camera.

### Data analysis

#### Biomechanical profile of the giant swing

Kinematic and dynamic variables were extracted from the motion capture such as the horizontal and vertical positions of the center of gravity (CG) of each segment and of the gymnast's model (in m), the angular velocity of the gymnast (ω_G_, in °/s), and the shoulder and hip flexion-extension angle (in degree). Herein a flexion of the shoulder joint refers to a decrease in the trunk-upper arm angle of the digitized model, which is in contrast to the clinical frontal shoulder flexion that generally corresponds to an increasing angle between the trunk and the arm. The moment of inertia (*I*_G_, in kg.m^2^) around the gymnast's CG and the gymnast's total body energy (*E*_Tot_, in Joule/kg) were calculated. The moment of inertia around the gymnast's CG was computed as follows:

(1) $$I_{G}=\sum_{i=1}^{5}\left[I_{i}+(M\;\cdot \;m_{i})\;\cdot \;d_{i}^{2}\right],$$

where *I*_i_ was the moment of inertia of the *i*th segment, *M* the mass of the gymnast, *m*_i_ the mass of the *i*th segment, *d*_i_ the distance between the CG of the *i*th segment and the CG of the whole gymnast's body. According to de Leva (1996), the moment of inertia of each segment *i* was equal to:

(2) $$I_{i}=(M\;\cdot \;m_{i})\;\cdot \;{\left(l_{i}\;\cdot \;r_{i}\right)}^{2},$$

where *l*_i_ was the length of the *i*th segment and *r*_i_ was the radius of gyration of the *i*th segment about the transversal axis expressed as a proportion of the segment length.

According to Arampatzis and Brüggemann (2001), the gymnast's total body energy was equal to:

(3) $$E_{\text{Tot}}=\sum_{i\;=\;1}^{5}(1/2\;\cdot \;(M\;\cdot \;m_{i})v_{i}^{2}+1/2\;\cdot \;I_{i}\omega_{i}^{2}+(M\;\cdot \;m_{i})gh_{i}),$$

where *v*_i_ was the linear velocity of the *i*th segment, ω_i_ the angular velocity of the *i*th segment, *g* the acceleration due to gravity, and *h*_i_ the height of the *i*th segment center of gravity. *E*_Tot_ was normalized to the body mass of the gymnast for comparison purpose. The examined variables (positions of the CG of the gymnast, joint angles, angular velocity, moment of inertia, and mechanical energy) were presented as a function of the body position angle, from 0 to 360° with 0° corresponding to the vertical axis below the high bar.

#### Extraction of muscle synergies

As inter-cycle variability contains important information for identifying the muscle synergies (Clark et al., 2010; Ting et al., 2012), they were extracted from a set of nine consecutive giant swings, with the first and last giant swing being automatically removed. EMG signals were band-pass filtered (20–450 Hz, Butterworth filter, 2nd order), rectified, smoothed with a zero lag low-pass filter (9 Hz, Butterworth filter, 2nd order), and time-normalized in order to obtain 200 data points for each giant swing. EMG was normalized to the maximum level of activity across all giant swings (Turpin et al., 2011a). Therefore, as is classically done in studies focusing on muscle synergies, the degree of muscle activity was not taken into consideration.

NMF was performed from this dataset. For this purpose, we implemented the Lee and Seung (2001) algorithm. Matrix factorization minimizes the residual Frobenius norm between the initial matrix and its decomposition, and is given as:

(4) E=WC+e

(5) $$\underset{\begin{array}{c} W\;\geq \;0\\ C\;\geq \;0 \end{array}}{\min}\text{​​}\|{E-WC\|}_{\text{FRO}}$$

where **E** is a *p*-by-*n* initial matrix (*p* = number of muscles and *n* = number of time points), **W** is a *p*-by-*s* matrix (*s* = number of synergies), **C** is a *s*-by-*n* matrix, and *e* is a *p*-by-*n* matrix. ‖•‖_FRO_ establishes the Frobenius norm, **W** represents the muscle synergy vectors matrix, **C** is the synergy activation coefficients matrix, and **e** is the residual error matrix. The algorithm is based on iterative updates of an initial random guess of **W** and **C** that converge to a local optimal matrix factorization [see Lee and Seung (2001) for more details]. To avoid local minima, the algorithm was repeated 20 times for each subject. The lowest cost solution was kept (i.e., minimized squared error between original and reconstructed EMG patterns). The initial matrix **E** consisted of 9 consecutives giant swings for the 12 muscles. As each giant swing was interpolated to 200 time points, **E** was a 12-row and 1800-column matrix.

We iterated the analysis by varying the number of synergies between 1 and 12 and then selected the least number of synergies that accounted for >90% of variance accounted for (VAF) (Torres-Oviedo et al., 2006) or until adding an additional synergy did not increase VAF by >5% of VAF (Clark et al., 2010). Mean total VAF was defined as (Torres-Oviedo et al., 2006):

(6) $$\text{VAF}=1-\frac{\sum_{i\;=\;1}^{p}\sum_{j\;=\;1}^{n}{\left(e_{i,\;j}\right)}^{2}}{\sum_{i\;=\;1}^{p}\sum_{j\;=\;1}^{n}{\left(E_{i,\;j}\right)}^{2}}$$

As the determination of the correct number of muscle synergies is not a trivial matter (Tresch et al., 2006), we further confirmed our results by using the best linear fit (BLF) method which selected the smallest *n* such that a linear fit of the “VAF” vs. “number of synergies” curve, from *n* to 12, had a residual mean square error of less than 5 × 10^−5^ [i.e., the point at which the VAF curve plateaus to a straight line; see Cheung et al. (2005); Ajiboye and Weir (2009)]. Finally, we used a method reported by Cheung et al. (2009) named “knee point (KP)” herein. Briefly, the “VAF” vs. “number of synergies” curve was constructed from both the original EMG dataset and an unstructured EMG dataset generated by randomly shuffling the original dataset across time and muscles. *n* was then defined as the point beyond which the original-slope drops below 75% of the surrogate-slope. This corresponds to the number beyond which any further increase in the number of extracted synergies yields a VAF increase smaller than 75% of that expected from chance.

We calculated VAF for each muscle (VAF_muscle_) to ensure that each muscle activity pattern was well accounted for by the extracted muscle synergies [for further details, see Hug et al. (2011)]. Finally, to further determine the subject-specific dimensionality of the data we calculated, for each gymnast, VAF for each of the extracted muscle synergies.

#### Cross-validation of the extracted muscle synergies

To verify the within-subject consistency of the extracted muscle synergies, we used a cross-validation procedure as proposed by previous research (e.g., Cheung et al., 2005, 2009; Ting and Chvatal, 2010). For each participant, we checked that the muscle synergy vectors extracted for one set of giant swings (first set) accounted for the EMG patterns in the other set. To do this, the muscle synergy matrix (muscle synergy vectors) was held fixed in the algorithm and the coefficients matrix was free to vary [for additional details, see Hug et al. (2011)].

#### Between-subject similarity

The comparison of the shape (i.e., waveform) of mechanical patterns, individual EMG patterns and synergy activation coefficients was assessed using two criterions: the Pearson's correlation coefficient (*r*) and the circular cross-correlation coefficient (*r*_max_). We also calculated the absolute lag times that assess differences in the timing of the activations (i.e., the magnitude of the time shift between mechanical patterns, EMG patterns or between synergy activation coefficients) as the lag time at the maximum of the cross-correlation function. As we are aware of the fact that *r*-values, *r*_max_-values and lag times provide some redundant information, we chose to report all of these indexes to increase our ability to compare our results with other studies that did not necessary report all these types of information. The index of similarity corresponded to both the averaged *r*- and *r*_max_-value between each pair of participants.

We then determined the similarity of muscle synergy vectors across participants by calculating a Pearson's correlation coefficient between each pair of participants. Based on the same principle that was previously described by Safavynia and Ting (2012), we considered a pair of muscle synergy vectors to be similar if *r* = 0.71, which corresponds to the critical value of *r* for 10 degrees of freedom (i.e., 12 − 2 muscles) at *p* = 0.01. However, because the NMF algorithm constrains muscle weightings to be non-negative, one would expect positive correlation by chance (Safavynia and Ting, 2012). Therefore, for each extracted synergy we generated 1000 random permutations of the weightings obtained from the extraction of the muscle synergy vectors. Then we calculated the *r*-value for each pair (36 pairs × 1000 iterations = 3600 *r*-values) and for each synergy, yielding a distribution of *r*-values expected by chance. An *r*-value of 0.71 corresponded to the 99th percentile of the distribution. Consequently, we considered a pair of muscle synergy vectors with a *r* = 0.71 more similar than expected by chance, and thus muscle synergy vectors with a *r* < 0.71 were considered different.

To further assess the similarity of the muscle synergies between the gymnasts, we checked that the muscle synergies extracted from one gymnast accounted for the overall and individual EMG patterns of each of the other gymnasts. The first step aimed at identifying the robustness of the muscle synergy vectors across the subjects. To do this, the muscle synergy vectors matrix extracted from one subject (i.e., control subject herein, **W**_control_) was held fixed in the NMF algorithm while the activation coefficient matrix of the compared subject (**C**_subject_) was free to vary (Torres-Oviedo et al., 2006; Hug et al., 2011). **C**_subject_ was initialized with random values and iteratively updated until convergence. The EMG data matrix of the compared subject (**E**_subject_) was provided to the algorithm with the following update rule (Lee and Seung, 2001):

(7) $${\left({\text{C}}_{\text{subject}}\right)}_{ij}\leftarrow {\left({\text{C}}_{\text{subject}}\right)}_{ij}\frac{{\left({\text{W}}_{\text{control}}{\text{E}}_{\text{subject}}\right)}_{ij}}{{\left({\text{W}}_{\text{control}}{\text{W}}_{\text{control}}{\text{C}}_{\text{subject}}\right)}_{ij}}$$

This process was performed for each of the 72 pairwise comparisons (nine gymnasts compared with the eight others). The overall VAF and VAF_muscle_ were used to quantify the success of the fixed muscle weightings and the newly computed synergy activation coefficients to reconstruct the EMG patterns. A VAF_muscle_ > 75% was considered satisfying (Torres-Oviedo and Ting, 2007). The second step was similar to the first but aimed to determine the robustness of the activation coefficients across the participants by fixing the activation coefficients matrix (**C**_control_) while the muscle synergy vectors matrix (**W**_subject_) was free to vary. Finally, a Two-Way ANOVA (factors: muscles and reconstruction methods, i.e., fixed muscle synergy vectors vs. fixed synergy activation coefficients) was used to determine whether VAF_muscle_ differed between the muscles and was influenced by the reconstruction method (fixed vectors vs. fixed coefficients). *Post-hoc* analyses were made with Scheffe's tests. The level of significance was *p* = 0.05.

## Results

### Mechanical data

Figure 2 depicts the kinematic and dynamic data computed from the motion capture of the giant swings. Due to the hip and the shoulder flexion, the gymnasts managed to increase their angular velocity (on average from 230 to 275°/s between 35 and 90° of the giant swing), which when associated with the decrease in the moment of inertia of the gymnast, allowed an increase in mechanical energy to a sufficient level to attain the handstand position above the bar (i.e., 180° of the giant swing).

Relative to the horizontal and vertical positions of the CG of each segment of the gymnast's model, the indices of similarity (i.e., *r* and *r*_max_) were extremely high, ranging from 0.98 ± 0.02 to 1.00 ± 0.00. Regardless of the height of the participants, the trajectory of the CG of the gymnast's model was similar among them (Figure 2) with an averaged *r*-value and *r*_max_-value of 1.00 ± 0.00. The averaged absolute lag time between each pair of participants was below 1% of the giant swing for each kinematic parameter (horizontal and vertical position of the segments' and gymnast's CG). The indices of similarity for the shoulder and hip joint angles, the angular velocity, the moment of inertia, and the mechanical energy of the gymnast are reported in Table 1. Except for the moment of inertia that exhibited a low averaged *r*-value (0.32 ± 0.55 and range: −0.81 to 0.97) due to large time shifts (averaged lag time = 17.2 ± 17.3%; range: 0.5–50.0% of the giant swing), all the biomechanical variables were similar among the participants. The lag that leads to differences in moment of inertia was mainly attributable to participant #7 who exhibited a moment of inertia in anti-phase relative to the other participants. This confirms that our population of gymnasts was homogeneous as they performed their backward giant swings similarly in terms of kinematics as well as dynamics.

**Table 1:** **Similarity of the kinematic and dynamic parameters across participants**.

	***r***	***r*_max_**	**Lag (%)**
Shoulder angle	0.83 (0.36–0.96)	1.00 (1.00–1.00)	4.1 (0.0–11.5)
Hip angle	0.88 (0.62–0.98)	1.00 (1.00–1.00)	2.1 (0.0–5.5)
Angular velocity	0.97 (0.95–0.99)	1.00 (0.99–1.00)	0.9 (0.0–2.5)
Moment of inertia	0.32 (-0.81–0.97)	1.00 (0.99–1.00)	17.2 (0.5–50.0)
Mechanical energy	0.89 (0.77–0.97)	1.00 (0.99–1.00)	2.2 (0.0–4.5)

Values are mean (min-max). Lags were calculated as the lag times that maximized the cross-correlation function and correspond to the absolute time shift between the two waveforms (% giant swing).

Except for the moment of inertia that exhibited a low averaged r-value due to large time shifts (about 17% of the giant swing), all the biomechanical variables were similar among the participants.

### Individual EMG patterns

**Figure 3:**
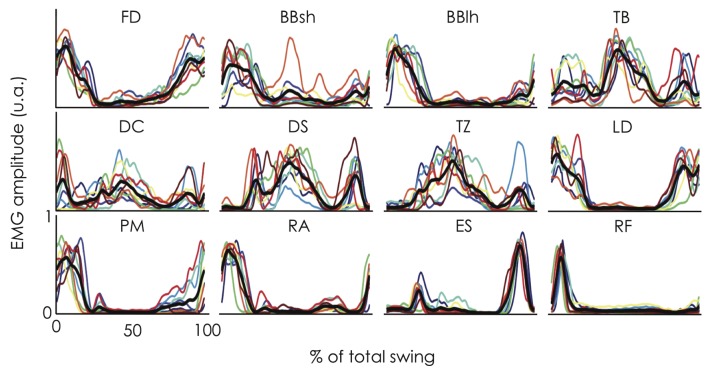
**Electromyographic (EMG) envelope for 12 muscles obtained in nine gymnasts during linked backward giant swings.** Each profile represents an individual EMG pattern averaged across 9 consecutive giant swings and is expressed as a function of the percentage of the giant swing (0 and 100% correspond to the vertical position of the gymnast under the bar). The black bold line indicates the mean profile across the nine gymnasts while the thin color lines represent the individual data (for the color legend, see Figure 1). EMG was normalized to the maximum level of activity across all giant swings. While the pattern of activity of some muscles exhibited high similarity between participants (e.g., FD, BBlh, LD, RA, ES, and RF), others were more variable (e.g., BBsh, TB, DC, DS, and TZ). Variability between participants can be partly explained by time shifts of the EMG patterns. FD, flexor digitorum; BBsh, short head of the biceps brachii; BBlh, long head of the biceps brachii; TB, lateral head of the triceps brachii; DC, clavicular part of the deltoideus; DS, scapular part of the deltoideus; TZ, upper part of the trapezius; LD, latissimus dorsi, PM, sternocostal part of the pectoralis major; RA, rectus abdominis; ES, erector spinae at level of L4; RF, rectus femoris.

For each participant, the EMG patterns for the 12 muscles investigated are depicted in Figure 3. The inter-subject indices of similarity (i.e., *r* and *r*_max_) are reported in Table 2. The averaged *r*-value between each pair of participants was 0.70 ± 0.20, and ranged from 0.26 (DC) to 0.89 (FD). The averaged *r*_max_-value was 0.90 ± 0.05, and ranged from 0.83 (DC) to 0.96 ± 0.02 (FD and RA). While the pattern of activity of some muscles exhibited high similarity between participants (e.g., FD, BBlh, LD, RA, ES, and RF), others were more variable (e.g., BBsh, TB, DC, DS, and TZ). The higher *r*_max_-values compared with *r*-values showed that the variability between participants can be partly explained by time shifts of the EMG patterns. Indeed, we found an absolute lag time ranging from 1.5% (ES) to 18.5% (DC) in the giant swing (Table 2). The largest time shifts were observed for BBsh, DC, and TB muscles and were mainly attributable to participant #6 and #7 (Figure 3).

**Table 2:** **Between-subject variability of the EMG profiles**.

	***r***	***r*_max_**	**Lag (%)**
Flexor digitorum	0.89 (0.75–0.96)	0.96 (0.92–0.99)	2.1 (0.0–6.0)
Biceps brachii (short head)	0.55 (0.03–0.96)	0.85 (0.67–0.98)	13.3 (0.0–40.5)
Biceps brachii (long head)	0.80 (0.44–0.98)	0.93 (0.83–0.99)	3.2 (0.0–8.5)
Triceps brachii	0.52 (0.04–0.90)	0.86 (0.71–0.97)	9.0 (0.0–46.0)
Deltoideus (anterior part)	0.26 (–0.37–0.81)	0.83 (0.71–0.94)	18.5 (0.0–48.0)
Deltoideus (posterior part)	0.58 (0.02–0.83)	0.84 (0.66–0.94)	5.4 (0.0–46.0)
Trapezius	0.59 (0.14–0.91)	0.86 (0.67–0.96)	5.8 (0.0–24.0)
Latissimus dorsi	0.82 (0.49–0.96)	0.92 (0.81–0.99)	2.9 (0.0–12.0)
Pectoralis major	0.74 (0.49–0.98)	0.90 (0.73–0.99)	4.1 (0.0–10.0)
Rectus abdominis	0.86 (0.51–0.99)	0.96 (0.89–0.99)	2.4 (0.0–7.5)
Erector spinae	0.87 (0.58–0.98)	0.95 (0.89–0.99)	1.5 (0.0–4.0)
Rectus femoris	0.86 (0.46–0.97)	0.94 (0.85–1.00)	1.6 (0.0–4.5)

Values are mean (min-max). Lags were calculated as the lag times that maximized the cross-correlation function and correspond to the absolute time shift between the two waveforms (% giant swing).

While the pattern of activity of some muscles exhibited high similarity between participants (e.g., FD, BBlh, LD, RA, ES, and RF), others were more variable (e.g., BBsh, TB, DC, DS, and TZ). As r_max_-values are very high, variability between participants can be partly explained by time shifts of the EMG patterns.

### Number of extracted muscle synergies

**Figure 4:**
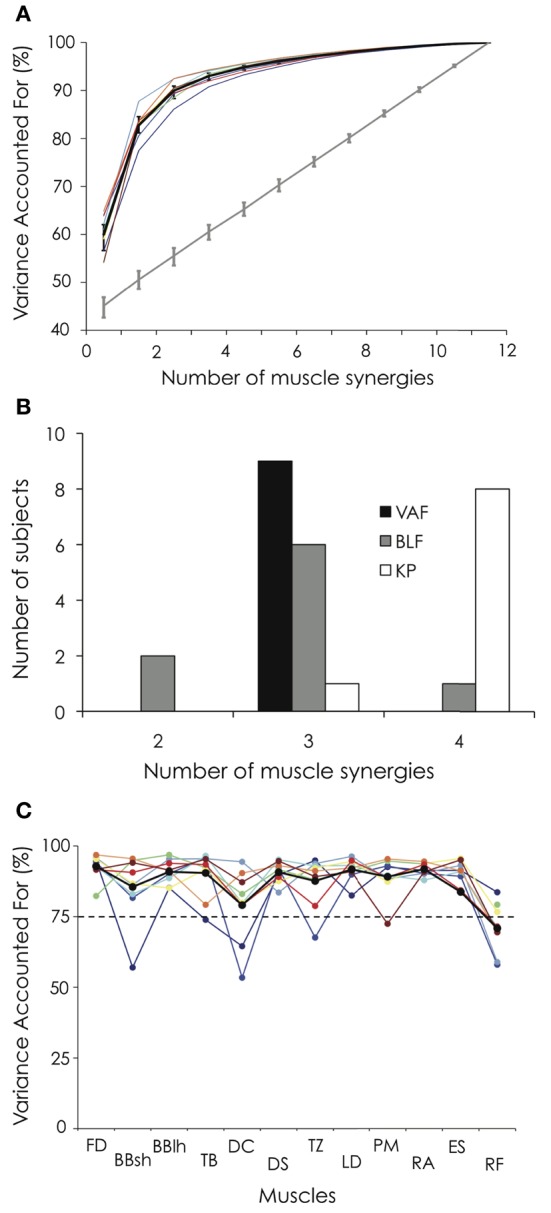
**Variance accounted for (VAF) and number of extracted muscle synergies. (A)** The percentage of variance accounted for is depicted for each participant as a function of the number of extracted synergies. Error bars indicate the 95% bootstrap confidence interval across the participants for both the VAF calculated from the original data set (black bold line) and the VAF calculated from the unstructured EMG dataset generated by randomly shuffling the original dataset across time and muscles (gray bold line). **(B)** Number of extracted muscle synergies based on the VAF threshold method (VAF), the best linear fit method (BLF, Cheung et al., 2005) and the knee point method (KP, Cheung et al., 2009). **(C)** VAF_muscle_ is depicted for each participant and each muscle. For both Panels (**A** and **C**), the black bold line indicates the mean profile across the nine gymnasts. Abbreviations for individual muscles are described in the legend of Figure 3. For the color legend, see Figure 1.

Figure 4A depicts the cumulative percentage of variance explained by each number of muscle synergies. Using the criterion previously described (i.e., VAF > 90% or until adding an additional synergy did not increase VAF by >5%), three synergies were identified for all the participants. When applying the BLF method, 6 out of 9 participants exhibited 3 muscle synergies (Figure 4B). When applying the KP method describing by Cheung et al. (2009), 8 out of 9 participants exhibited 4 muscle synergies (Figure 4B). These three analyses reveal that all (or most of) the participants exhibited the same number of muscle synergies (100% for the threshold method, 66% for BLF, and 89% for KP). Because it has not been demonstrated that one of this methods is more accurate than another to determine the correct number of muscle synergies and because three muscle synergies were found to characterize the data in 2 out of the 3 methods, we decided to use three muscle synergies for all the participants for the subsequent analysis.

**Figure 5:**
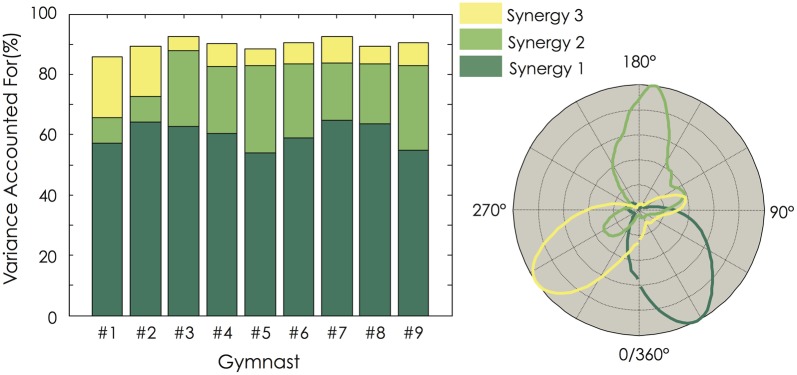
**Variance Explained For (VAF) for each of the three extracted muscle synergies.** For each gymnast, VAF explained by each of the three extracted muscle synergies was calculated (**Left panel**). The temporal activation of each muscle synergy is depicted as a function of the mean angular position (**Right panel**). For all the gymnasts, it clearly appears that the dimensionality in their EMG data was mainly explained by the first muscle synergy. Gymnasts #1 and #2 exhibited a higher VAF by the third synergy than the second one. This strategy differs from the seven other gymnasts.

Three muscle synergies accounted for a mean VAF of 89.9 ± 2.0% (range: 86.1–92.5%) and the VAF_muscle_ ranged from 70.9 ± 8.5 to 92.8 ± 4.3% (Figure 4C). While VAF_muscle_ of BBsh, DC, and TZ was lower than 75% for some participants (1–2, depending on the muscle), VAF_muscle_ consistently dropped below 75% for RF. The VAF explained by each of the three extracted muscle synergies is depicted in Figure 5 for each gymnast. We observed between-subject variability, especially for synergy #2 and #3 (coefficient of variation = 6.3, 34.7, and 53.9% for synergy #1, #2, and #3, respectively). This variability can be explained mainly by the fact that VAF was higher for synergy #3 compared to synergy #2 for participant #1 and #2, while it was to the contrary for all the other participants (Figure 5).

### Within-subject consistency of the extracted muscle synergies

**Figure 6:**
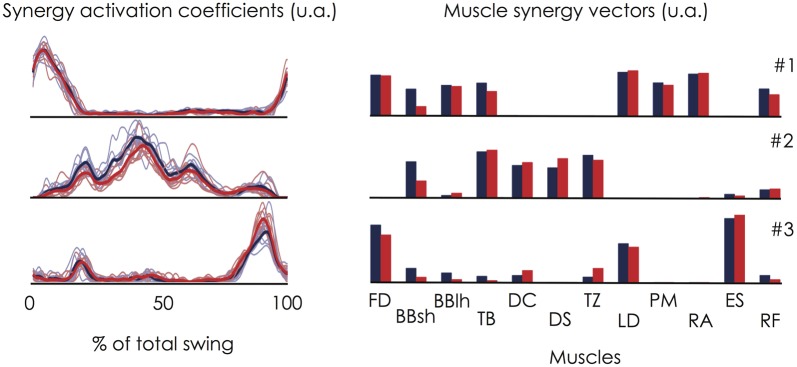
**Within-subject consistency of the muscle synergies extracted during the two sets of linked backward giant swings.** This figure depicts an individual example (Participant #6). On the left panel, the thin lines correspond to the synergy activation coefficient extracted for each giant swing and the bold lines correspond to the averaged profile over the 9 consecutive giant swings. Red stands for the set #1 and blue stands for the set #2. The corresponding muscle synergy vectors are depicted on the right panel. This figure clearly shows that the muscle synergies were robust for a given participant. Indeed, the cross-validation procedure showed that the muscle synergy vectors extracted for the first set of linked backward giant swings explained 87.9 ± 2.7% (range: 83.5–91.7%) of the variability of the dataset obtained during the second set. Abbreviations for individual muscles are described in the legend of Figure 3.

An individual example (participant #6) of the three muscle synergies extracted during both the first and the second set is depicted in Figure 6. The cross-validation procedure showed that the muscle synergy vectors extracted for the first set of linked backward giant swings explained 87.9 ± 2.7% (range: 83.5–91.7%) of the variability of the dataset obtained during the second set. To further assess the repeatability of the extracted muscle synergies, we compared the two sets of giant swings. Both the synergy activation coefficients and the muscle synergy vectors exhibited good repeatability. The averaged *r*-value over the three muscle synergies was 0.93 ± 0.05 (range: 0.88–0.98) for the synergy activation coefficients and 0.93 ± 0.06 (range: 0.87–0.98) for the muscle synergy vectors.

Overall, these results clearly show that the muscle synergies were robust for a given participant allowing us to interpret a difference between participants as different motor control strategies rather than as methodological issues.

### Between-subject variability of the extracted muscle synergies

**Figure 7:**
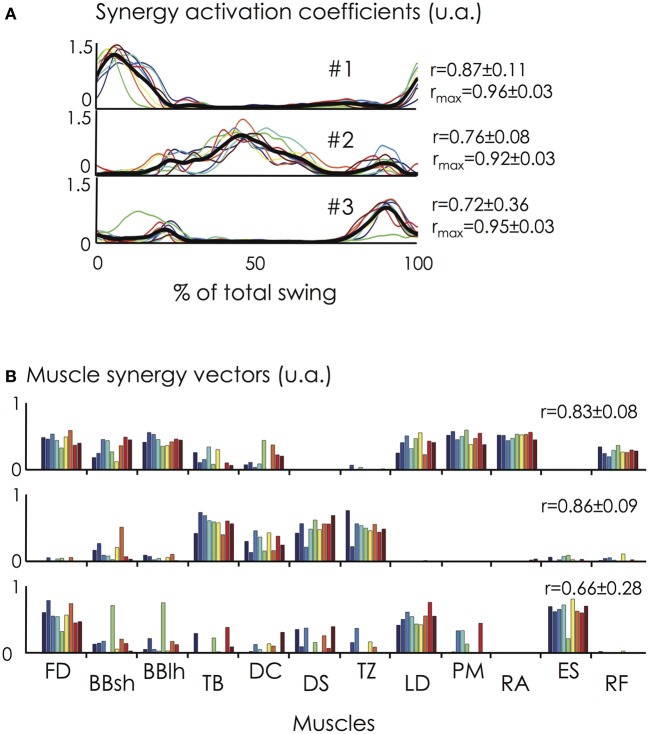
**Synergy activation coefficients (*C*) and muscle synergy vectors (*W*) across the nine gymnasts. (A)** The synergy activation coefficients are shown at the top of the figure for all the participants and for the three extracted synergies (solid lines in different colors). The synergy activation coefficients are expressed as a function of the percentage of the giant swing (0 and 100% correspond to the vertical position of the gymnast under the bar). The mean synergy activation coefficient over all the participants is represented by the black bold line. **(B)** The muscle synergy vectors are shown at the bottom of the figure for all the participants. Individual muscle weightings are depicted for each muscle within each synergy. *r*- and/or *r*_max_-values correspond to the averaged value between each pair of participants. Synergy #1 mainly involved trunk and hip flexor muscles (e.g., LD, PM, RA, RF) at the beginning of the ascendant phase of the swing. Synergy #2 mainly involved arm (TB) and shoulder muscles (DC, DS, TZ) and was activated during the upper part of the giant swing. Finally, synergy #3 mainly involved FD, ES, and LD and is activated to ensure the grip on the bar and the hip extension of the gymnast. For muscle abbreviations, see the Figure 3 legend. For the color legend, see Figure 1.

The three extracted muscle synergies are depicting in Figure 7. The temporal activation of muscle synergies (i.e., synergy activation coefficients) was consistent across participants [*r*-value of 0.87 (range: 0.53–0.98), 0.76 (range: 0.50–0.87), and 0.72 (range: −0.03–0.98) for synergy #1, #2, and #3, respectively; *r*_max_-value of 0.96 (range: 0.87–0.99), 0.92 (range: 0.86–0.98), and 0.95 (range: 0.85–0.99) for synergy #1, #2, and #3, respectively]. The higher *r*_max_-values compared with *r*-values suggest that variability between participants is partly explained by time shifts. The mean absolute lag time was 4.3 ± 2.1% of the giant swing and ranged from 2.7 ± 1.9% (synergy #1) to 6.7 ± 9.9% (synergy #3) of the giant swing. The larger time shift observed in synergy #3 compared to synergy #1 and #2 was mainly attributable to participant #5. Indeed, its peak of activation occurred during the first half of the swing (<50% of the total swing), while the other participants had their peak of activation coefficients in the second half of the swing (Figure 7).

Concerning the muscle synergy vectors, we found an averaged *r*-value of 0.83 (range: 0.62–0.97), 0.86 (range: 0.64–0.98), and 0.66 (range: 0.03–0.97) for synergy #1, #2, and #3, respectively. Considering the critical *r*-value of 0.71 (see methods), four pairwise comparisons (out of 36 possibilities, i.e., 11%) were different for synergy #1, two (6%) were different for synergy #2, and 13 were different for synergy #3 (36%). This clearly shows that the composition of synergy #1 and #2 was consistent across participants while the composition of synergy #3 was more variable, as highlighted by Figure 7.

**Figure 8:**
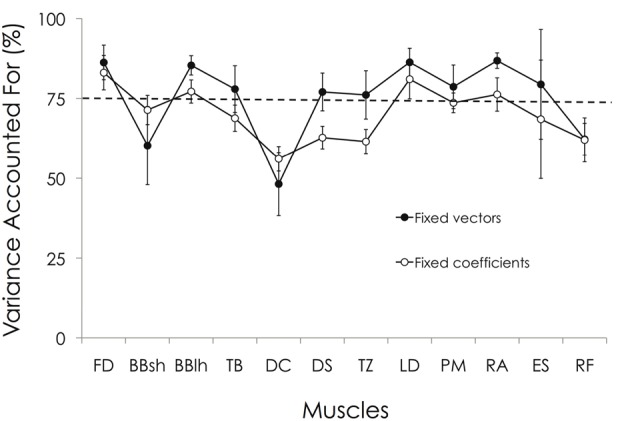
**VAF_muscle_ obtained by keeping either the muscle synergy vectors constant or the synergy activation coefficients.** Two additional analyses have been performed to test the similarity of the muscle synergies between participants (see “Materials and Methods” section). First, by keeping the muscle synergy vectors constant, we obtained on averaged a VAF_muscle_ ranging between 48.2 ± 9.9% (DC) and 86.8 ± 2.4% (RA). Relative to the preset threshold of VAF_muscle_ > 75%, the EMG patterns of BBsh, DC, and RF muscles were not correctly reconstructed. By keeping the synergy activation coefficients constant, the VAF_muscle_ ranged between 56.1 ± 3.8% (DC) and 83.0 ± 5.4% (FD). Relative to the preset threshold of VAF_muscle_ > 75%, the EMG pattern of BBsh, TB, DC, DS, TZ, PM, ES, and RF muscles were not correctly reconstructed. For muscle abbreviations, see Figure 3 legend.

As explained in the Methods, two additional analyses have been performed to test the similarity of the muscle synergies between participants. First, by keeping the muscle synergy vectors constant, we obtained an averaged VAF across all pairs of 79.3 ± 3.7% (range: 70.6–87.5%). The VAF_muscle_ ranged between 48.2 ± 9.9% (DC) and 86.8 ± 2.4% (RA). Relative to the preset threshold of VAF_muscle_ >75%, the EMG patterns of the BBsh, DC, and RF muscles were not correctly reconstructed (Figure 8). By keeping the synergy activation coefficients constant, the averaged VAF was 72.4 ± 4.8% (range: 60.2–82.9%). The VAF_muscle_ ranged between 56.1 ± 3.8% (DC) and 83.0 ± 5.4% (FD). Relative to the preset threshold of VAF_muscle_ >75%, the EMG pattern of the BBsh, TB, DC, DS, TZ, PM, ES, and RF muscles were not correctly reconstructed (Figure 8). The Two-Way ANOVA showed a significant main effect for both “muscle” and “reconstruction method” (*p* < 0.01). More precisely, VAF_muscle_ was significantly lower when the synergy activation coefficients were fixed than when muscle synergy vectors were fixed (70.2 ± 10.5% vs. 75.4 ± 14.1%, respectively). VAF_muscle_ was significantly lower for BBsh, DC, DS, TZ, and RF muscles than for the others. Overall, these results suggested that the muscle synergy vectors were more consistent across the gymnasts than the synergy activation coefficients.

## Discussion

The results of the present study outlined three muscle synergies that accounted for the EMG patterns during giant swings on a high bar. The relative consistency of muscle synergies across trained gymnasts confirms that muscle synergies are consistent across participants (Torres-Oviedo and Ting, 2007; Cheung et al., 2009; Hug et al., 2011; Turpin et al., 2011b; Chvatal and Ting, 2012), even during a skilled motor task requiring learning.

### Methodological considerations

As done in previous research (Turpin et al., 2011a), EMG activity from each muscle was normalized to its peak value from all of the cycles. Note that this normalization procedure only provides information about the level of muscle activity in relation to this peak value (i.e., waveform of the EMG patterns). In other words, while interindividual variability in terms of degree of muscle activity can exist, the present study only focuses on the EMG waveform variability. This choice was motivated by the fact than an ideal normalization method to quantify the degree of muscle solicitation does not exist (Burden, 2010). Whatever the normalization method, a part of the observed variability would have been attributable to methodological considerations. Consequently, we considered a muscle synergy as a covariation of muscle activation where the output level of this activation was not taken into consideration.

By quantifying the interindividual variability using *r*-values, *r*_max_-values, and lag times, our goal was to compare our results with those of the literature. However, it should be kept in mind that the smoothing of both the EMG patterns and the synergy activation coefficients, can influence the *r*-values (Hug, 2011). As a wide variety of low-pass filters have been used in the literature aimed at extracting muscle synergies during locomotor tasks, e.g., from 4 Hz in Clark et al. (2010) to 40 Hz (Chvatal and Ting, 2012), caution must be taken when comparing the results of interindividual variability from studies that used different cut-off frequencies.

### Functional roles of muscle synergies

The extracted muscle synergies were well related to the mechanics of the giant swing. Synergy #1 mainly involved the trunk and hip flexor muscles (e.g., LD, PM, RA, RF) at the beginning of the ascendant phase of the swing that would allow the gymnast to decrease his moment of inertia, and gain some mechanical energy and angular velocity to attain the handstand position. The forearm muscle (FD) was also involved in synergy #1 to firmly grip the bar [which was highly in tension in that phase of the swing (Cagran et al., 2010)] while arm (BBsh, BBlh) muscles were solicited to stiffen elbow and glenohumeral joints, respectively. Synergy #2 mainly involved the arm (TB) and shoulder muscles (DC, DS, TZ) and was activated during the upper part of the giant swing. In light of the inverse dynamic model of a ground handstand (Kerwin and Trewartha, 2001), synergy #2 was activated to support the body weight. The activation profile of synergy #2 also showed a second lower peak near the end of the descendant phase of the giant swing, simultaneously with the peak in activity for synergy #3 and with the peak in angular velocity. The angular velocity of the gymnast increased due to the gravitational acceleration up to this peak, which might coincide with the end of the “fall-like” part of the giant swing and with the highest tensile load on the high bar (Cagran et al., 2010). Therefore, the arm and shoulder muscles of synergy #2 (TB, DC, DS, TZ), plus the trunk muscles of synergy #3 (LD) were activated to limit the extension and the tensile load within the shoulder joint. Finally, the other muscles of synergy #3 ensured the grip on the bar (FD) and hip extension (ES) of the gymnast's body. This arch-like position of the body would set the tension in the flexor chain muscles and favor the subsequent shoulder flexion during the ascending section (Frère et al., 2012).

### Interindividual variability of the neuromuscular control strategies

Interindividual variability in EMG patterns has often been reported at the level of individual muscles (Ryan and Gregor, 1992; Guidetti et al., 1996; Hug and Dorel, 2009; Hug et al., 2011). It is also the case in the present study where some individual EMG patterns (e.g., BBsh, TB, DC, DS, and TZ) exhibited interindividual variability that seems to be higher than the variability reported during pedaling (Hug et al., 2010). This difference may be explained by several factors, such as the number of degrees of freedom (closed vs. open kinematic chain for pedaling and giant swing, respectively) and the higher smoothing of the EMG profiles in the study by Hug et al. (2010), which may increase the similarity of the waveform (Hug et al., 2012).

It is unclear whether this variance in muscle activation across subjects would arise from variance in the motor program itself. In some cases, different muscle synergies have been identified in subpopulations (Torres-Oviedo and Ting, 2007). For instance, Torres-Oviedo and Ting (2007) extracted in some participants a muscle synergy specific to a knee-bending strategy during balance control. In contrast, despite a relatively high interindividual variability of some individual muscles, Hug et al. (2010) reported similar modular organization of muscle coordination (in terms of number of extracted muscle synergies, composition, and temporal activation) across trained cyclists during pedaling. In the present study, three consistent muscle synergies accounted for the EMG patterns in trained gymnasts during a giant swing, as reported in other cyclic tasks such as pedaling and rowing (Hug et al., 2010; Turpin et al., 2011b). Despite the overall similarity of both muscle synergy vectors and synergy activation coefficients across gymnasts, some differences occurred (36% of the pairwise comparisons of muscle synergy vectors), mainly for synergy #3. As this synergy is activated at the end of the descendant phase, the variability of the muscle synergy vectors may be explained by a lower muscular demand. Indeed, during the descendant phase of the giant swing, muscular torque accounted for less than gravitational and inertial torques to enable the arch-like position of the gymnast (Sevrez et al., 2012). According to previous studies demonstrating that the spatial components of the muscle synergies are related to biomechanical functions (Ting and Macpherson, 2005; Torres-Oviedo and Ting, 2007; McKay and Ting, 2008), this low muscular demand might involve subtle subject-specific muscle synergy compositions. High tensile load was determined at the end of the descending phase of the giant swing (Cagran et al., 2010). To counteract this tensile load, gymnasts stiffened the shoulder joint likely by the second peak of activity visible in synergy #2 rather than by synergy #3. This may confirm the relationship between muscle synergy composition and functional demand. This also confirms previous observations that although some muscle synergies are very robust across subjects, others are more variable (Hug et al., 2010).

A key bit of information provided by the synergy analysis regards the number of extracted muscle synergies that have been proposed to reflect the complexity of motor control (Clark et al., 2010). As justified in the Methods section, we extracted the same number of muscle synergies for all the participants. However, the determination of the correct number of muscle synergies is not a trivial matter (Tresch et al., 2006) and despite the use of different criterion, we cannot affirm that all the participants exhibit the same number of muscle synergies and thus that they exhibit the same complexity of motor control. However, the low coefficient of variations (mean/SD × 100) in VAF values (ranging from 0% for 12 muscles synergies to 7% for 1 muscle synergies; 2.2% for 3 muscle synergies) highly suggests that the gymnasts possess a very similar dimensionality in their EMG data.

### Neurophysiological interpretations

The present results showed a strong similarity in neuromuscular control strategies across the experts during a skilled motor task (Figure 7). This consistency in muscle synergies might reflect the existence of lower-level neural control structures that can be flexibly modulated to result in complex, learned movements as previously suggested (Cheung et al., 2005; Ting and McKay, 2007; Torres-Oviedo and Ting, 2007; Hug et al., 2011; Chvatal and Ting, 2012). During skill learning, it has been shown that the modulation of muscles synergy composition emerged up to a stable state allowing a subsequent change in the temporal profile of the muscle synergies (Kargo and Nitz, 2003). This suggests that muscle synergies may be formed by adaptive processes in relationship to the experiences of each individual. Consequently, the relatively good similarity of muscle synergies observed between the gymnasts could be explained by their similar training experience. It should also be noted, however, that instead of constructing new muscle synergies during the learning process, it is also possible that the extracted muscle synergies have been adapted from existing synergies (Safavynia et al., 2011). Although numerous studies have suggested that the central nervous system produces movement through a flexible combination of muscle synergies (Ting and McKay, 2007), it should be kept in mind that other research has suggested that the synergies better reflect task constraints (Kutch et al., 2008; Valero-Cuevas et al., 2009). Therefore, the consistency observed in the present study might also be explained by the mechanical requirements demanded by the task and would only signify that the observed synergies are compatible with the execution of a backward giant swing. As we studied only one condition (without varying constraints), we were not able to test this hypothesis. However, although mechanical constraints were similar across individuals, high interindividual variability was evident for some EMG patterns (e.g., DS, TZ, TB, Figure 3), confirming that different muscle activity patterns may lead to similar mechanical patterns, or task performance (Chvatal et al., 2011).

The higher VAF and VAF_muscle_ values obtained when muscle synergy vectors were fixed compared to fixed coefficients of activation further suggest that muscles synergies are spatially fixed while their temporal patterns of recruitment can vary (Chvatal and Ting, 2012; Safavynia and Ting, 2012). This spatial consistency of the nervous control of motor behavior might support the notion that descending cortical signals represent neuronal drives that select, activate, and flexibly combine muscle synergies specified to networks in the spinal cord and/or brainstem (Hart and Giszter, 2004; Cheung et al., 2005). In this way, it has been shown that only the temporal activation of muscle synergies (and not the spatial structure) is altered by deafferentation or cortical stroke in humans (Cheung et al., 2005, 2009).

## Conclusion

Although variability was found (especially for synergy #3), the gymnasts exhibited gross similar neuromuscular strategies when performing several consecutive giant swings. This confirms that muscle synergies are consistent across participants, even during a skilled motor task requiring learning. Further investigations are necessary to both confirm that these muscle synergies reflect lower-level neural control rather than biomechanical constraints and to understand whether they are constructed during the learning process or whether they have been adapted from existing synergies.

### Conflict of interest statement

The authors declare that the research was conducted in the absence of any commercial or financial relationships that could be construed as a potential conflict of interest.
